# Pleural effusion within 24 h of admission predicts severe hypertriglyceridemia-induced acute pancreatitis and organ failure: retrospective cohort of 296 patients

**DOI:** 10.3389/fmed.2026.1757038

**Published:** 2026-01-23

**Authors:** Ziyu Liu, Yuxian He, Zhuo Chen, Meiru Liu, Huihong Zhai

**Affiliations:** Department of Gastroenterology, Xuanwu Hospital, Capital Medical University, Beijing, China

**Keywords:** early predictor, hypertriglyceridemia-induced acute pancreatitis, organ failure, pleural effusion, severity

## Abstract

**Background:**

Early risk-stratification in hypertriglyceridemia-induced acute pancreatitis (HTG-AP) is challenging within the first 24 h of admission. We assessed whether pleural effusion (PE) detected on early imaging serves as an early predictor for the development of severe HTG-AP (HTG-SAP) and organ failure (OF), compared with BISAP, MCTSI, and C-reactive protein (CRP).

**Methods:**

A retrospective study included 296 HTG-AP patients at Xuanwu Hospital from August 2013 to February 2024. PE presence within 24 h was abstracted from chest CT; outcomes were HTG-SAP and OF. Diagnostic performance was evaluated using ROC analysis and DeLong tests, comparing with BISAP, MCTSI, and CRP. The prognostic impact of PE laterality (unilateral vs. bilateral) was also analyzed.

**Results:**

PE demonstrated the strongest positive correlation with hypertriglyceridemia-induced severe acute pancreatitis (HTG-SAP) (OR: 6.82; 95% CI: 3.13–14.88; *p* < 0.001). PE achieved the area under the curve (AUC) of 0.792 (95% CI: 0.742–0.837) for HTG-SAP prediction and 0.718 (95% CI: 0.663–0.768) for OF anticipation. The predictive accuracy of PE was significantly higher than that of CRP for predicting HTG-SAP (ΔAUC = 0.106; *p* = 0.004) and comparable for predicting OF (ΔAUC = 0.055; *p* = 0.112). There was no significant difference between PE and MCTSI scores in predicting either HTG-SAP or OF. However, PE had a lower predictive accuracy compared with the BISAP for both HTG-SAP (ΔAUC = 0.057) and OF (ΔAUC = 0.083; *p* < 0.001). Despite this, PE still demonstrated substantial predictive value for HTG-SAP and OF. There was no significant difference between unilateral and bilateral pleural effusions in predicting the risk of developing HTG-SAP and OF.

**Conclusion:**

PE detected within 24 h is a simple, readily available early radio-graphic marker that identifies patients at high risk for progressing to severe course and OF in HTG-AP. While not superior to BISAP, PE offers pragmatic utility when composite scores or complete laboratories are unavailable and may trigger early monitoring and escalation.

## 1 Introduction

Hypertriglyceridemia (HTG) is a well-established cause of acute pancreatitis (AP), and the incidence of hypertriglyceridemia-induced AP (HTG-AP) is rising globally (1). HTG-AP is more prone to progress to severe acute pancreatitis (SAP) with multiorgan dysfunction (2–4). SAP carries a mortality rate exceeding 30%, contributing to significantly higher morbidity and mortality than moderately severe AP (MSAP). The initial 24-h period following hospital admission is regarded as a critical juncture for the management of SAP, wherein timely intervention can exert a substantial influence on prognosis (5). Nevertheless, the early (< 24 h) assessment of AP severity remains a clinical challenge, and no satisfactory methodology has yet been established. Given the increased risk of severe progression in HTG-AP, early and accurate prediction of SAP risk is essential for timely ICU referral and targeted intervention (6). The implementation of such strategies is expected to reduce mortality, and complications associated with HTG-AP, thereby improving patient outcomes.

Currently, four major scoring systems are widely utilized for the early identification of SAP: the Acute Physiologic and Chronic Health Evaluation (APACHE II), Ranson score, Bedside index of AP severity (BISAP), and modified CT severity index (MCTSI). However, each system exhibits notable limitations in clinical practice. BISAP and MCTSI are more widely used in clinical settings due to their simplicity. The BISAP score, while simple and applicable within 24 h, exhibits limited sensitivity (65%) for predicting SAP (7, 8). The organ specificity of MCTSI improves its accuracy, but it needs to be graded according to local complications and requires more than 72 h to evaluate (9). Additionally, individual laboratory markers like red blood cell distribution width (RDW), serum calcium (Ca^2+^), and C-reactive protein (CRP) (10) is commonplace, yet these markers often lack sufficient sensitivity or specificity (1, 11). Therefore, advancing scoring methods is still needed to enhance predictive accuracy (12).

Pleural effusion (PE) is a prevalent complication of AP and has been identified as an early indicator of disease severity (13, 14). It has been particularly predictive of SAP within the first 48 h of hospital admission (15). A meta-analysis revealed that patients with AP and PE have a higher risk of developing complications such as pancreatic pseudocysts, accelerated pancreatic necrosis, and increased mortality compared to those without PE (16). Despite this, most existing studies on PE and AP outcomes have included heterogeneous etiologies and lack specific analyses within the HTG-AP population— a group characterized by more severe systemic inflammation and faster disease progression. Unlike previous studies that often used a 48-h window for PE detection or mixed AP subtypes, we specifically focus on the HTG-AP subtype and evaluate whether pleural effusion detected within 24 h of admission can serve as an early imaging biomarker for predicting progression to SAP and organ failure (OF) in this study. By narrowing the etiological spectrum and standardizing the imaging assessment window, we aim to determine whether early pleural effusion can facilitate more accurate and timely risk stratification in HTG-AP.

## 2 Materials and methods

### 2.1 Study design and participants

**FIGURE 1:**
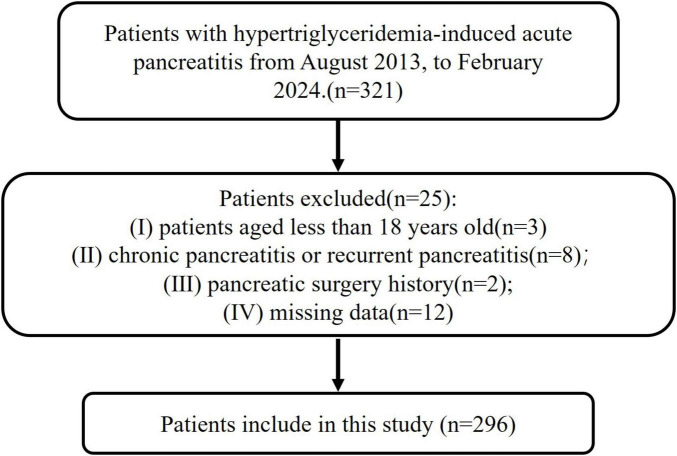
Flow chart for inclusion and exclusion of patients. Patients (totally 321) with hypertriglyceridemia-induced acute pancreatitis (HTG-AP) from 1 August 2013, to 15 February 2024. A total of 25 people were excluded based on the exclusion criteria. The exclusion criteria included (I) age less than 18 years old; (II) recurrent AP and chronic pancreatitis; (III) cases with missing data; and (IV) pre-existing pleural effusion or pleural effusion caused by other causes. A total of 296 patients were finally included in this study.

Patients admitted and treated in the gastroenterology department of Xuanwu Hospital from August 2013 to February 2024 with confirmed diagnosis of HTG-AP were all enrolled into this study. The exclusion criteria were as follows: (1) age less than 18 years old; (2) recurrent AP and chronic pancreatitis; (3) cases with missing data; and (4) pre-existing pleural effusion or pleural effusion caused by other causes (cirrhosis and hypoproteinemia, cancer, congestive heart failure, pneumonia and pulmonary embolism, etc.) (17). In all, 321 HTG-AP patients were initially identified and 25 of them were excluded because of the exclusion criteria. Thus, 296 HTG-AP patients were included in this study (Figure 1). This study was approved by the Ethics Committee of the Xuanwu Hospital of Capital Medical University and written informed consent was waived considering the retrospective study design.

### 2.2 Definitions

The diagnostic criteria for HTG-AP required fulfillment of either: serum triglyceride levels ≥ 11.3 mmol/L, or levels between 5.65 and 11.3 mmol/L accompanied by lipemic serum and ≥ 2 of the following: (1) typical pancreatitis-related abdominal pain, (2) serum amylase and/or lipase activities exceeding threefold the upper normal limit, and (3) imaging evidence of acute pancreatitis (18). HTG-AP severity was classified using 2012 revision of the Atlanta classification (19), which categorizes patients into three tiers based on OF status and complications: mild acute pancreatitis (HTG-MAP) was defined by the absence of organ failure and local/systemic complications; moderately severe acute pancreatitis (HTG-MSAP) involved transient OF (resolving within 48 h), localized complications, or acute exacerbation of preexisting comorbidities; and severe acute pancreatitis (HTG-SAP) was characterized by persistent OF (exceeding 48 h). OF was evaluated using the modified Marshall scoring system, with failure defined as a score ≥ 2 in any of the following: respiratory, cardiovascular, and renal systems. Prognostic thresholds were defined by 2012 revision of the Atlanta classification: BISAP ≥ 3, MCTSI ≥ 4 and CRP ≥ 150 mg/L indicates a poor clinical prognosis.

### 2.3 Data collection

The collected general data comprised 5 categories of data: demographic variables, vital signs, laboratory findings, imaging results, and prognostic indicators. Demographic variables encompassed gender, age, body mass index (BMI), and medical history of metabolic syndrome such as diabetes mellitus, hypertension, hyperlipidemia. Vital signs recorded at admission included heart rate (HR), respiratory rate (RR), systolic and diastolic blood pressure. Laboratory findings performed within 24 h of admission consisted of triglycerides (TG), white blood cell (WBC), red cell distribution width (RDW), neutrophil-lymphocyte ratio (NLR), albumin (ALB), blood urea nitrogen (BUN), serum creatinine (Cr), Serum calcium (Ca^2+^), CRP, D-dimer, procalcitonin (PCT). Imaging results performed within 24 h of admission included chest CT (uniformly performed for all patients) and chest X-ray (used as an auxiliary tool only when CT was temporarily unavailable), with specific attention to the presence of PE. According to the recommendations of the Fleischner Society (20), pleural effusion was defined as a homogeneous and peripheral opacification free of any air bronchograms and characterized by a CT attenuation lower than the CT attenuation of adjacent alveolar consolidation. On X-ray, this included blunting of the costophrenic angles, meniscus sign, and fluid in the fissures. Any detectable fluid on chest CT was considered positive for PE, regardless of volume. All imaging studies were systematically reviewed by a single radiologist with over 10 years of experience in thoracic imaging, who was blinded to clinical data, patient outcomes, and laboratory results. Prognostic indicators tracked until discharge or clinical endpoint included clinical outcomes like occurrence of SAP, OF, systemic inflammatory response syndrome (SIRS), clinical scoring systems such as BISAP (0–5), and MCTSI (0–10), and hospital day.

### 2.4 Statistical analysis

Continuous variables were assessed for normal distribution using the Shapiro–Wilk test and were expressed as mean ± standard deviation and analyzed via independent samples *t*-tests. Non-normally distributed variables were characterized by median (interquartile range) values and subjected to Mann–Whitney U tests and Kruskal–Wallis H tests. Categorical data were summarized as frequencies with percentages and evaluated through χ^2^ tests or Fisher’s exact probability tests, as appropriate for count distributions. Subsequently, Variables with *p* < 0.05 in baseline data comparisons and some other important clinical variables were entered into univariable and multivariable logistic regression analyses for further analyses. Exclude variables with collinearity before performing multivariate logistic regression analysis. Multivariable logistic regression analyses were performed to identify independent risk factors for HTG-SAP and OF. Variables with *p* < 0.05 in univariable analyses and clinically relevant covariates were entered into multivariable models. Multicollinearity was assessed using the variance inflation factor (VIF), and variables with VIF > 5 were excluded before model fitting. Odds ratios (ORs) with 95% confidence intervals (CIs) were reported. All statistical computations were conducted utilizing IBM SPSS Statistics software (version 26.0; IBM Corp). Diagnostic performance assessments were subsequently implemented through receiver operating characteristic (ROC) curve analysis employing MedCalc Software (v20.113), with area under the curve (AUC) comparisons executed to quantify the predictive accuracy of prognostic indicators (PE, CRP, BISAP, MCTSI) for HTG-SAP and OF development. The sensitivity and specificity corresponding to the best cut-off value were calculated. Statistical equivalence of ROC curves (ΔAUC) was assessed via Z-tests based on DeLong’s method. A two-tailed α-level of *p* < 0.05 was defined as the threshold for statistical significance.

## 3 Results

### 3.1 General characteristics of the HTG-MAP, HTG-MSAP and HTG-SAP groups

According to the inclusion criteria and exclusion criteria 296 HTG-AP patients were enrolled in this study. Baseline clinical characteristics, stratified by the grade of HTG-AP, are presented in Table 1. The incidence of PE was significantly higher in the HTG-SAP patients (75%), compared with HTG-MAP (33%) and HTG-MSAP (4%) (*p* < 0.001). Several variables were positively correlated with increased disease severity, including age, HR, RR, specific laboratory markers (TG, WBC, RDW, NLR, ALB, CRP, d-dimer and PCT) and prognostic indicators (organ failure, SIRS, CRP ≥ 150, BISAP ≥ 3, MCTSl ≥ 4, hospital day). In contrast, Ca^2+^ (2.23 ± 0.21 vs. 2.18 ± 0.25 vs. 2.01 ± 0.44; *p* < 0.001) were significantly lower in HTG-SAP patients compared to those without HTG-SAP. No other variables showed significant differences between the groups (Table 1).

**TABLE 1:** Baseline characteristics of participants categorized by HTG-AP severity.

Patient characteristics	HTG-MAP (*n* = 131)	HTG-MSAP (*n* = 98)	HTG-SAP (*n* = 67)	*P*-value
**Demographic variables**				
Gender, *N* (%)				0.282
Male	109 (83%)	75 (77%)	50 (75%)	
Female	22 (17%)	23 (23%)	17 (25%)	
Age, years	40 ± 15	37 ± 10	40 ± 16	***0.005***
BMI, kg/m^2^	27.68 ± 4.9	28.31 ± 5.58	28.28 ± 7.36	0.167
Diabetes, *N* (%)	61 (47%)	40 (41%)	27 (40%)	
Hypertension, *N* (%)	44 (34%)	30 (31%)	13 (19%)	0.111
Hyperlipidemia, *N* (%)	92 (70%)	70 (71%)	40 (60%)	0.228
**Vital signs**				
HR, bpm	85 ± 19	98.5 ± 24.25	109 ± 26	***0.000***
RR, bpm	19 ± 5	21 ± 5	25.207 ± 6.033	***0.000***
**Laboratory findings**				
TG, mmol/L	19.33 ± 13.53	20.81 ± 13.7	23.47 ± 12.72	***0.010***
WBC, *10^9^/L	10.59 ± 5.09	12.6 ± 5.54	13.79 ± 5.65	***0.000***
RDW, fL	12.6 ± 1	12.9 ± 0.83	13 ± 1.2	***0.008***
NLR	6.07 ± 4.87	7.1 ± 6.8	12.678 ± 7.117	***0.000***
ALB, g/L	43.05 ± 7.14	40.85 ± 9.19	40.49 ± 8.54	***0.001***
Cr, μmol/L	58 ± 19	57 ± 24	81.149 ± 78.005	0.595
Ca^2+^, mmol/L	2.23 ± 0.21	2.18 ± 0.25	2.01 ± 0.44	***0.000***
CRP, mg/L	78 ± 106.7	167.5 ± 168.48	234 ± 216	***0.000***
D-dimer, μg/L	0.44 ± 0.68	1.05 ± 1.92	1.36 ± 2.6	***0.000***
PCT, ng/mL	0.09 ± 0.16	0.23 ± 0.5	1.415 ± 2.285	***0.000***
**Imaging results**				
Pleural effusion, *N* (%)	5 (4%)	32 (33%)	50 (75%)	***0.000***
**Prognostic indicators**				
Organ failure	4 (3%)	26 (27%)	65 (97%)	
SIRS	47 (36%)	76 (78%)	64 (96%)	***0.000***
CRP ≥ 150, (mg/L)	27 (21%)	55 (56%)	49 (73%)	***0.000***
BISAP ≥ 3, *N* (%)	0 (0%)	0 (0%)	3 (4%)	***0.006***
MCTSI ≥ 4, *N* (%)	4 (3%)	43 (44%)	47 (70%)	***0.000***
Hospital day, days	11 ± 5	15 ± 8	20.28 ± 8.65	***0.000***

HTG-MAP, hypertriglyceridemia-induced mild acute pancreatitis; HTG-MSAP, hypertriglyceridemia-induced moderate severe acute pancreatitis; HTG-SAP, hypertriglyceridemia-induced severe acute pancreatitis; HR, heart rate; RR, respiratory rate; TG, triglyceride; WBC, white blood cell; RDW, red cell distribution width; NLR, neutrophil-lymphocyte ratio; ALB, albumin; Cr, serum creatinine; Ca^2+^, serum calcium; CRP, C-reactive protein; PCT, procalcitonin; SIRS, systemic inflammatory response syndrome; BISAP, bedside index for severity in acute pancreatitis; MCTSI, modified CT severity index. The bold values indicate *P* < 0.05, signifying statistical significance.

### 3.2 Univariable and multivariable logistic regression

Initial screening of 25 baseline variables identified 12 significant predictors (*p* < 0.05), including age, HR, RR, TG, WBC, RDW, NLR, ALB, Ca^2+^, CRP, D-dimer, and PE. Given the established association between metabolic syndrome components and HTG-AP severity (21, 22), diabetes mellitus and hyperlipidemia history were additionally incorporated, yielding 14 candidate variables for multivariate analysis. Multivariable logistic regression revealed 7 independent predictors of HTG-SAP (Table 2): Age (OR: 1.05; 95% CI: 1.01–1.09; *p* = 0.022), RR (OR: 1.14; 95% CI: 1.06–1.24; *p* = 0.001), TG (OR: 1.07; 95% CI: 1.02–1.12; *p* = 0.009), WBC (OR: 1.17; 95% CI: 1.05–1.29; *p* = 0.003), Ca^2+^ (OR: 0.11; 95% CI: 0.02–0.51; *p* = 0.005), d-dimer (OR: 1.22; 95% CI: 1–1.48; *p* = 0.045), the presence of PE (OR: 6.82; 95% CI: 3.13–14.88; *p* < 0.001). Notably, PE demonstrated the strongest independent predictor association with HTG-SAP, exhibiting both the highest odds ratio (OR) and most statistically significant *P*-value. Presence of PE increased the odds of HTG-SAP approximately sevenfold.

**TABLE 2:** Univariable and multivariable logistic regression analyses of predictive factors for HTG-SAP in all subjects.

Patient characteristics	All (*n* = 296)	HTG-SAP (*n* = 67)			
		Univariable regression		Multivariable regression	
		OR (95% CI)	*P*-value	OR (95% CI)	*P*-value
**Demographic variables**					
Age, years	39 ± 13	1.02 (0.99–1.04)	0.282	1.05 (1.01–1.09)	***0.022***
**Medical history**					
Diabetes, *N* (%)	128 (43%)	0.86 (0.49–1.49)	0.580		
Hyperlipidemia, *N* (%)	202 (68%)	0.61 (0.35–1.08)	0.089		
**Clinical signs**					
HR, bpm	94 ± 27.75	1.05 (1.03–1.07)	**0.000**		
RR, bpm	21 ± 6	1.2 (1.13–1.28)	**0.000**	1.14 (1.06–1.24)	***0.001***
**Laboratory findings**					
TG, mmol/L	21.295 ± 13.465	1.06 (1.02–1.10)	**0.001**	1.07 (1.02–1.12)	***0.009***
WBC, × 10^9^/L	11.97 ± 5.8975	1.17 (1.09–1.26)	**0.000**	1.17 (1.05–1.29)	***0.003***
RDW, fL	12.8 ± 1	1.5 (1.06–2.14)	**0.024**		
NLR	7.15 ± 6.8525	1.09 (1.05–1.14)	**0.000**		
ALB, g/L	42.11 ± 7.845	0.94 (0.90–0.99)	**0.009**		
Ca^2+^, mmol/L	2.175 ± 0.27	0.02 (0.01–0.08)	**0.000**	0.11 (0.02–0.51)	***0.005***
CRP, mg/L	126.1 ± 177.575	1.01 (1.00–1.01)	**0.000**		
D-dimer, μg/L	0.675 ± 1.3625	1.48 (1.26–1.74)	**0.000**	1.22 (1–1.48)	***0.045***
**Imaging results**					
Pleural effusion, *N* (%)	87 (29%)	15.26 (7.94–29.33)	**0.000**	6.82 (3.13–14.88)	***0.000***

HTG-SAP, hypertriglyceridemia-induced severe acute pancreatitis; HR, heart rate; RR, respiratory rate; TG, triglyceride; WBC, white blood cell; RDW, red cell distribution width; NLR, neutrophil-lymphocyte ratio; ALB, albumin; Ca^2+^, serum calcium; CRP, C-reactive protein. The bold values indicate *P* < 0.05, signifying statistical significance.

### 3.3 Predictive analysis of developing HTG-SAP and OF

We found a strong association between PE within 24 h of disease onset and both the severity of HTG-AP and the development of organ failure. To evaluate its predictive value, we compared PE to other scoring systems and CRP levels in assessing HTG-AP severity and clinical outcomes. Table 3 presents the area under the curve (AUC), sensitivity, and specificity of PE, CRP, and different scoring systems for predicting HTG-AP severity and clinical outcomes.

**TABLE 3:** Receiver operating characteristic curve analysis of pleural effusion, C-reactive protein, BISAP and MCTSI for predicting severity and organ failure of HTG-AP.

Characteristics	AUC (95% CI)	Sensitivity (%; 95% CI)	Specificity (%; 95% CI)	Δ AUC	z-statistic	*P*-value
**HTG-SAP**						
Pleural effusion	0.792 (0.742–0.837)	74.63 (62.5–84.5)	83.84 (78.4–88.4)	–	–	–
CRP (mg/L)	0.687 (0.630–0.739)	73.13 (60.9–83.2)	64.19 (57.6–70.4)	0.1060	2.865	***0.0042***
BISAP	0.849 (0.803–0.888)	73.13 (60.9–83.2)	88.21 (83.3–92.1)	0.0566	3.711	***0.0002***
MCTSI	0.796 (0.745–0.840)	70.15 (57.7–80.7)	79.48 (73.7–84.5)	0.0035	0.109	0.9129
**Organ failure**						
Pleural effusion	0.718 (0.663–0.768)	58.95 (48.4–68.9)	84.58 (78.8–89.3)	–	–	–
CRP (mg/L)	0.662 (0.605–0.716)	66.32 (55.9–75.7)	66.17 (59.2–72.7)	0.0552	1.587	0.1124
BISAP	0.801 (0.751–0.845)	57.89 (47.3–68.0)	89.55 (84.5–93.4)	0.0831	4.888	***< 0.001***
MCTSI	0.736 (0.681–0.785)	75.79 (65.9–84.0)	64.68 (57.6–71.3)	0.0179	0.643	0.5203

HTG-SAP, hypertriglyceridemia-induced severe acute pancreatitis; CRP, C-reactive protein; BISAP, bedside index for severity in acute pancreatitis; MCTSI, modified CT severity index. The bold values indicate *P* < 0.05, signifying statistical significance.

**FIGURE 2:**
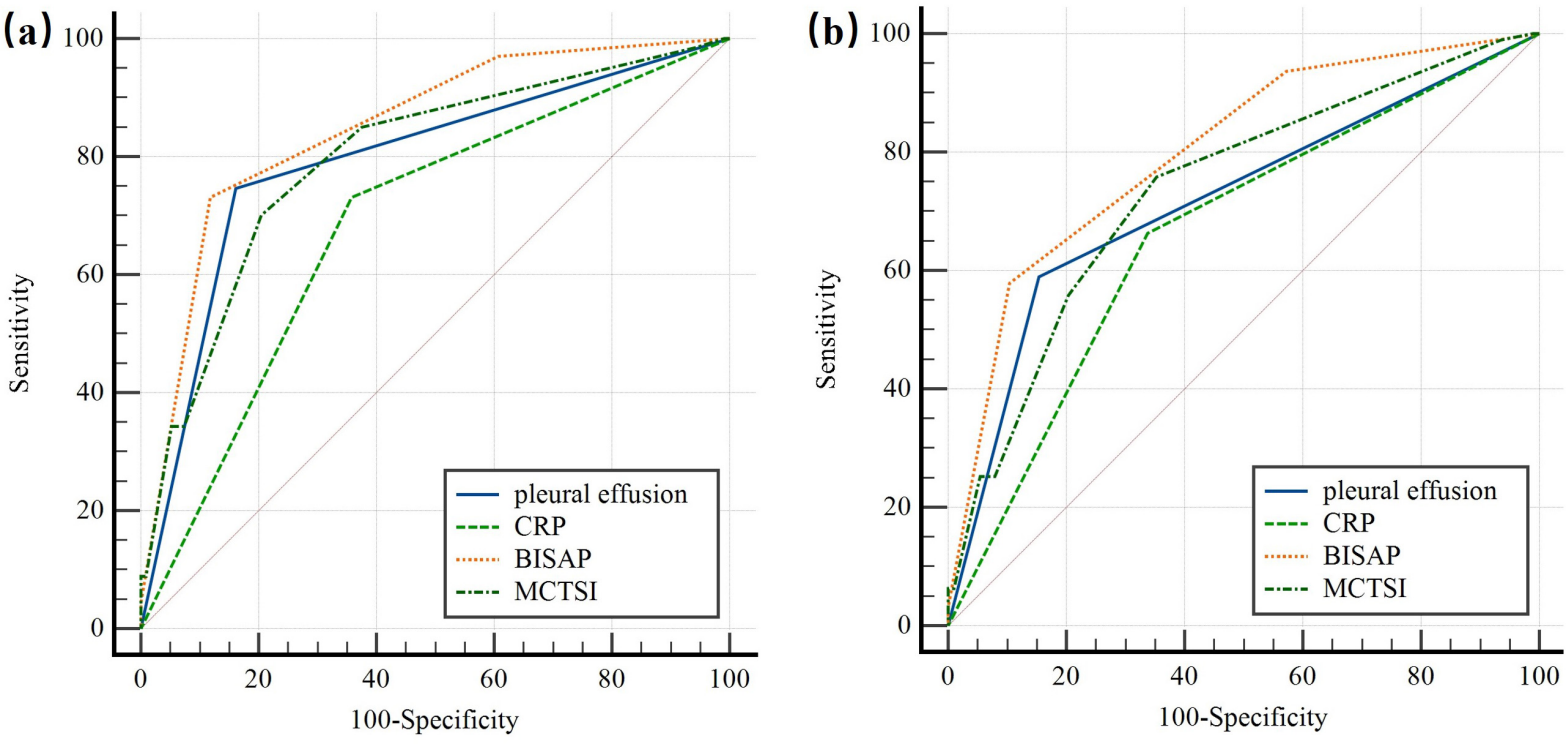
ROC curve of the pleural effusion volume, C-reactive protein levels and different clinical scoring systems for predicting **(a)** severe acute pancreatitis; **(b)** organ failure. The predictive accuracy of PE was comparable to that of CRP, and MCTSI in both HTG-SAP and organ failure. But PE had significantly lower accuracy than the BISAP score. PE: pleural effusion (blue); CRP: C-reactive protein (green); BISAP: bedside index of AP severity (orange); MCTSI: the Computed Tomography Severity Index (dark green).

For predicting HTG-SAP (Figure 2a), the AUC of PE was 0.792 (95% CI: 0.742–0.837; *p* < 0.001), with a sensitivity of 74.63% (95% CI: 62.5–84.5) and specificity of 83.84% (95% CI: 78.4–88.4). PE’s predictive accuracy was significantly better than CRP (ΔAUC = 0.106; *p* = 0.0042), and comparable to that of MCTSI (ΔAUC = 0.0035; *p* = 0.9129) scores, but significantly lower than the BISAP score (ΔAUC = 0.0566, *p* = 0.0002).

For predicting OF (Figure 2b), the AUC of PE was 0.718 (95% CI: 0.663–0.768; *p* < 0.001), with a sensitivity of 58.95% (95% CI: 48.4–68.9) and a specificity of 84.58% (95% CI: 78.8–89.3). The predictive accuracy of PE was comparable to CRP levels (ΔAUC = 0.0552; *p* = 0.1124), and MCTSI scores (ΔAUC = 0.0179; *p* = 0.5203). However, PE had significantly lower accuracy than the BISAP score (ΔAUC = 0.0831; *p* < 0.001).

### 3.4 PE side for evaluating the severity of HTG-AP, SIRS and OF

The study investigated the differences in the risk of adverse clinical outcomes between HTG-AP patients with unilateral and bilateral pleural effusion. Among patients with PE, 51.7% (45/87) had unilateral effusions including 37.9% (33/87) at left-sided and 13.8% (12/87) at right-sided, while 48.3% (42/87) had bilateral effusions.

Table 4 summarizes the correlation between pleural effusion distribution and clinical outcomes of HTG-AP. No significant correlation was found between the location of PE and clinical outcomes (e.g., CRP, CTSI, SIRS, OF) (*p* > 0.05).

**TABLE 4:** Correlation between unilateral and bilateral PE and the risk of adverse clinical outcomes.

Characteristics	PE side		*P*-value
	Unilateral (*n* = 45)	Bilateral (*n* = 42)	
HTG-SAP	22 (48.9%)	28 (66.7%)	0.094
CRP ≥ 150, (mg/L)	35 (77.8%)	30 (71.4%)	0.496
MCTSI ≥ 4	30 (66.7%)	31 (73.8%)	0.467
SIRS	39 (86.7%)	35 (83.3%)	0.663
Organ failure	27 (60.0%)	29 (69.0%)	0.379

HTG-SAP, hypertriglyceridemia-induced severe acute pancreatitis; CRP, C-reactive protein; MCTSI, modified CT severity index; SIRS, systemic inflammatory response syndrome.

## 4 Discussion

In this single-center cohort of patients with HTG-AP, PE detected within 24 h of admission was independently associated with a severe clinical course and organ failure. As an early, readily obtainable imaging sign, PE demonstrated discriminatory performance comparable to the MCTSI and superior to CRP, while remaining inferior to BISAP for predicting both HTG-SAP (ΔAUC = 0.0566) and organ failure (ΔAUC = 0.0831; both *p* < 0.001). These findings support the pragmatic role of PE as a simple “red-flag” marker that can inform early triage and monitoring when composite scores or complete laboratory results are not yet available. Prior multinational cohorts have linked PE to worse outcomes in acute pancreatitis overall, but most did not stratify by etiology and included only a small proportion of HTG-AP, limiting applicability to this subgroup (23–26). Etiology-specific analyses are important because HTG-AP often presents with higher inflammatory burden and earlier progression to severe disease. Our results extend existing evidence by focusing specifically on HTG-AP within a standardized 24-h imaging window and by directly comparing PE against commonly used tools (BISAP, MCTSI, CRP). We also deliberately excluded patients with chronic cardiopulmonary disease or concomitant pneumonia to reduce confounding from non-pancreatitis causes of pleural fluid, which may partially explain differences in effect sizes reported across cohorts.

Hyperlipidemia has now emerged as the second most common cause of AP globally, surpassing alcohol, and the prevalence in China of HTG-AP cases has been on a continuous rise over the past two decades attributed to socioeconomic and dietary factors (27). Studies have shown that compared with non-HTG-AP patients, HTG-AP patients have a higher proportion of males and are younger in age. HTG-AP patients also exhibit higher mortality, greater disease severity, and an increased recurrence rate (28). In our study, the robust association between early PE and HTG-SAP (OR: 6.82) may be attributed to the profound systemic inflammation characteristic of HTG-AP. The pathophysiological interplay between hypertriglyceridemia and acute pancreatitis involves the hydrolysis of excessive triglycerides by pancreatic lipase, leading to a surge in free fatty acids (FFAs). These FFAs induce direct acinar cell injury, intensify systemic inflammatory responses, markedly increases systemic vascular permeability, which facilitates fluid extravasation into body cavities, with the pleural space being a common site and contribute to microcirculatory disturbances, thereby predisposing patients to severe acute pancreatitis (SAP) with multiorgan dysfunction compared to other subtypes (29). Consequently, PE detected within the first 24 h serves as an early imaging indicator of a severe systemic inflammatory, heralding subsequent organ dysfunction.

Contrasting with Yan et al.’s (15) study, we deliberately excluded PEV as a predictive variable due to four methodological considerations. Firstly, volumetric measurement methods remain non-standardized in current clinical algorithms, with neither chest radiography (CXR) nor CT-based PEV models (e.g., ellipsoid/planimetry methods) have achieved consensus adoption under ACR/SIR guidelines (30). Secondly, specific quantitative criteria—such as fluid volume and characteristics—are not universally recognized or validated for predicting the severity of hyperlipidemic pancreatitis, making it challenging for clinicians to establish consistent and reliable assessment standards. This variability hampers the ability to develop unified clinical guidelines for evaluating the condition. Additionally, PEV assessment techniques are not widely implemented in primary care settings, and medical personnel frequently lack specialized training in its measurement. Furthermore, the absence of readily available software for accurate measurement further complicates the early recognition of critical conditions, potentially delaying timely referral to tertiary hospitals for further treatment.

The role of triglyceride levels in determining the severity of acute pancreatitis has been a subject of ongoing debate. Compared with the findings of Deng et al. (31), our findings further demonstrated that TG level was an independent high-risk factor for progression to severe HTG-AP (OR 1.07, *P* = 0.009). In contrast to the view held by some studies, which posit that TG acts more as a triggering factor rather than a determinant of severity, our results support a different perspective. This discrepancy may be attributed to variations in sample size and regional differences among study populations.

Supporting the association between TG levels and disease severity, a large retrospective study of 1,457 consecutive acute pancreatitis cases found that elevated TG was independently correlated with a more severe clinical course. The incidence of organ failure, persistent multiple organ failure, pancreatic necrosis, and acute collections all increased significantly with worsening severity of HTG (32). In another retrospective study involving 1,539 AP patients, Wan et al. (33) found that 461 individuals (30%) had elevated triglyceride levels, and the incidence of severe AP rose with increasing HTG severity. Among patients with severe and very severe HTG (*N* = 112), 29% developed acute necrotic collections and 35% developed pancreatic necrosis. These patients also exhibited higher rates of persistent organ failure, multiple organ failure, persistent systemic inflammatory response syndrome, and a greater need for ICU admission (33).

In addition, our study only investigated the relationship between unilateral or bilateral PE and the risk of HTG-SAP, and no significant correlation was found between the location of PE (unilateral or bilateral) and the development of HTG-SAP, SIRS, or OF (*p* > 0.05). There could be two main reasons for these results. First, since this study included only 296 patients, the small sample size may have led to different outcomes. Second, most pleural effusions associated with pancreatitis tend to occur on the left side, possibly due to anatomical proximity.

Spatial anatomical relationships further influence the laterality of PE. Pancreatic inflammation and diaphragmatic vulnerabilities predispose fluid migration toward the left thoracic cavity. Bao et al. (34) postulated that left-sided predominance may stem from the larger surface area of the left lumbar costal triangle and its proximity to the pancreas. Our findings align with this observation: bilateral PE occurred in 48.3% of cases (42/87), while left-sided PE was present at 37.9% (33/87) and right-sided PE at only 13.8% (12/87), underscoring a clear leftward bias. Some of the results in our study are consistent with those of some previous results (15, 35). For example, in the study by Yan et al. (15), the incidence of pleural effusion in AP patients was 49.9% (232/465), with 72.4% (168/232) being bilateral, 23.7% (55/232) on the left side, and 3.9% (9/232) on the right side (*p* < 0.001).

PE’s predictive ability is comparable to that of MCTSI (ΔAUC = 0.0035 and 0.0179 for predicting HTP-SAP and OF; *p* > 0.05). Since MCTSI assessment includes both pancreatic fluid collection and pancreatic necrosis, pancreatic fluid collection might not score highly on imaging at 24 h, while pancreatic necrosis typically only becomes apparent later in the disease course. This results in MCTSI having low accuracy during early evaluation, usually requiring 72 h for accurate assessment. While originally designed for mortality prediction, the BISAP score has been extensively validated in subsequent research as a reliable tool for early identification of Severe Acute Pancreatitis (SAP) and organ failure. Its predictive objective aligns directly with the primary clinical endpoint of our study—the development of SAP.

All components of the BISAP score can be obtained objectively within the first 24 h of admission, perfectly matching our study’s timeframe for evaluating early predictors. Its inclusion of dynamic criteria like SIRS already captures relevant pathophysiological processes. Although PE shows slightly lower predictive accuracy compared to the BISAP score (ΔAUC = 0.0566 and 0.0831 for predicting HTP-SAP and OF; *p* < 0.001), its use as a standalone indicator provides distinct advantages in clinical practice. The BISAP score includes five criteria, making it more complex to apply than pleural effusion. Additionally, some criteria in the BISAP score, such as altered mental status, rely on subjective judgment by clinicians, which may introduce assessment bias. The practical utility of PE lies in its simplicity and immediacy. In busy emergency departments or resource-limited settings where calculate a full BISAP score might be delayed due to pending laboratory results or the need for comprehensive clinical assessment, the presence of PE on a CT scan—often performed urgently to confirm the diagnosis of AP—provides an instantaneous, objective risk signal. This can prompt clinicians to prioritize these patients for intensive monitoring, aggressive fluid resuscitation, or early specialist consultation, even before a formal composite score is available.

This investigation acknowledges 6 primary limitations. First, as a single-center study, the findings are subject to regional, racial, and lifestyle influences, which may limit the generalizability of the results regarding HTG-AP. The single-center study and admission criteria may affect the representativeness of the sample. To enhance the robustness of the findings, future research should include a multicenter approach with diverse geographic and demographic populations. Second, low event rates (mortality < 8%) in this Grade III Level A hospital compromised statistical power for PE-mortality correlation analysis. Furthermore, the extended study period (2013–2024) may have introduced heterogeneity, such as imaging protocols, intensive care unit admission criteria, and treatment guidelines could have evolved over time. However, it is important to note that the core diagnostic criteria for HTG-AP, the use of early CT imaging for severity assessment, and the fundamental principles of supportive care remained largely consistent in our tertiary center throughout this period. The non-significant *P*-value for the comparison between unilateral and bilateral PE should be interpreted with caution due to potential limited statistical power in this subgroup analysis. Although standardized resuscitation protocols were followed, the timing and intensity of specific interventions (like fluid resuscitation or insulin/heparin therapy for HTG) during the first 24 h could have modified the clinical course and potentially affected the development of SAP. Larger prospective studies are needed to clarify the above two specific association. Lastly, in more remote or underdeveloped regions, access to diagnostic tools for detecting PE may be limited, reducing the practicality of using PE as an early predictor of disease severity in such areas.

## 5 Conclusion

In conclusion, early prediction and management of HTG-SAP remain critical challenges in clinical practice. This study demonstrates that PE detected within 24 h acts as a simple, readily available early warning sign, identifying HTG-AP patients at high risk for progressing to severe disease and organ failure. While inferior to BISAP for discrimination, it provides pragmatic utility for early triage when rapid scoring is not feasible.

## Data Availability

The raw data supporting the conclusions of this article will be made available by the authors, without undue reservation.
